# Modification of Nuclear Compartments and the 3D Genome in the Course of a Viral Infection

**DOI:** 10.32607/actanaturae.11041

**Published:** 2020

**Authors:** S. V. Razin, A. A. Gavrilov, O. V. Iarovaia

**Affiliations:** Institute of Gene Biology Russian Academy of Sciences

**Keywords:** nuclear compartmentalization, spatial organization of the eukaryotic genome, virus infection

## Abstract

The review addresses the question of how the structural and functional compartmentalization of the cell nucleus and the 3D organization of the cellular genome are modified during the infection of cells with various viruses. Particular attention is paid to the role of the introduced changes in the implementation of the viral strategy to evade the antiviral defense systems and provide conditions for viral replication. The discussion focuses on viruses replicating in the cell nucleus. Cytoplasmic viruses are mentioned in cases when a significant reorganization of the nuclear compartments or the 3D genome structure occurs during an infection with these viruses.

## INTRODUCTION

To date, there is little doubt that structural and functional compartmentalization of the cell nucleus plays an important role in the functioning of the genetic machinery. Moreover, the genome itself is a structural platform for nuclear compartmentalization [1]. Individual chromosomes occupy limited spaces within the nucleus, which are referred to as chromosome territories [2, 3, 4]. Although relatively isolated, chromosome territories form numerous interchromosomal contacts. In addition, they attach to the nuclear lamina and nucleolus, thus forming a single chromatin domain. This domain is permeated by interchromatin channels, which together constitute the interchromatin compartment [2-6]. Various functional centers, such as the nucleolus, Cajal bodies, PML bodies, speckles, and transcription factories, are located inside this compartment [1, 5, 6]. Although these functional centers, many of which are also called nuclear bodies, are located in the interchromatin compartment, it is wrong to assume that they lack DNA. DNA is found in transcription factories located in the so-called perichromatin region lining interchromatin channels [5, 6]. The nucleolus is a special form of transcription factory located around clusters of ribosomal genes [7]. Speckles and Cajal bodies are reaction centers in which post-transcriptional RNA modification takes place and the necessary enzymes accumulate [8, 9, 10]. DNA is not an integral part of these functional compartments. However, there is ample evidence that genes can be recruited to them during the processing of various RNAs [11 , 12, 13].

The highest levels of spatial organization of the genome in the cell nucleus are as follows: (i) spatial segregation of active (A) and inactive (B) genomic compartments [14]; (ii) separation of chromosomes into partially insulated topologically associating domains (TADs) [15, 16, 17], which in many cases limit the areas of enhancer action [18, 19, 20]; and (iii) the establishment of spatial interactions between distant genomic elements by looping of the segments of the chromatin fiber separating them [21]. The functional significance of these spatial contacts may vary. In mammalian cells, contacts between the convergent binding sites of the insulator protein CTCF separate TADs [22]. Spatial contacts between enhancers and promoters (enhancer-promoter loops) ensure communication between these regulatory elements [23]. Changes in the spatial organization of the genome, including those resulting from chromosomal rearrangements and loss of CTCF-binding sites, alter the transcription profiles. In some cases, these changes cause cancer and other diseases [18, 24-28].

As mentioned above, the packed genome is a platform for structural and functional compartmentalization of the cell nucleus. However, the opposite is also true. The interaction between certain genomic regions and functional nuclear compartments supports the 3D organization of the genome. Thus, spatial segregation of the A and B genomic compartments is due to the re cruitment of active genes to speckles and the relocation of repressed genes to the nucleolus and nuclear lamina [13, 29, 30, 31]. Recruitment of various genes to Cajal bodies and common transcription factories facilitates the establishment of spatial contacts between the distant regions of the genome, as well as between different chromosomes [11, 32-36].

Viruses replicating in the cell nucleus exploit cellular systems during the infectious process. Although the features of the infectious process differ significantly for different viruses and depend on the type of infection (lytic/latent), it is apparent that viruses must adapt functional compartmentalization of the nucleus to suit their needs. Although the interaction between a virus and the host cell has been studied for decades, this aspect of the problem has not yet received enough of researchers’ attention. In this review, an attempt is made to summarize current knowledge on how viruses modify the nuclear compartments and the 3D organization of the cell genome. Although our discussion mainly focuses on the viruses replicating in the cell nucleus, we will also mention cytoplasmic viruses, which somehow cause reorganization of either nuclear compartments or the 3D genome upon infection.

## REORGANIZATION AND REPROFILING OF PRE-EXISTING NUCLEAR COMPARTMENTS DURING A VIRAL INFECTION

**Fig. 1:**
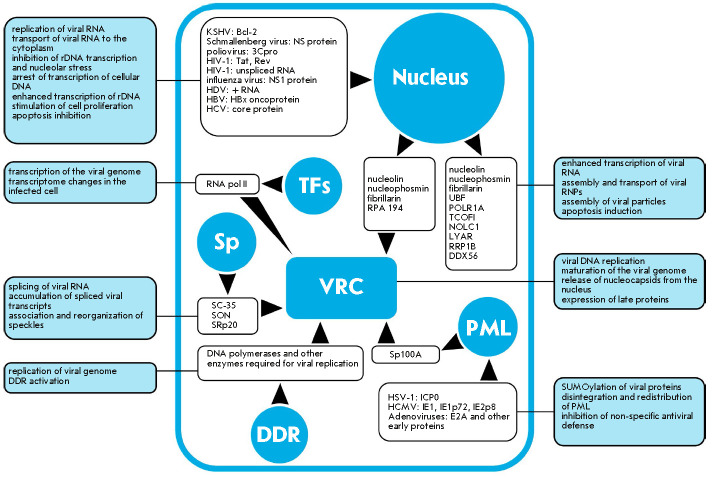
Scheme of movement of cellular and viral proteins/nucleic acids between nuclear compartments during infection. Blue circles indicate nuclear compartments: the nucleolus, transcription factories (TFs), speckles (Sp), promyelocytic leukemia (PML) bodies, DNA damage repair (DDR) foci, and viral replication centers (VRCs). Within the nucleus there are viral/cellular proteins and nucleic acids that move during the infectious process. Directions of movement are marked with black arrows. The rectangles with rounded corners contain information on the effects on cellular and viral metabolism associated with the movement of proteins/nucleic acids to/from the corresponding compartment during the infections process

Many nuclear compartments are modified during a viral infection (*Fig. 1*). These modifications happen because viruses need to either suppress the cellular antiviral defense or use the enzymes that have accumulated in the compartments for their replication. Viruses control the reorganization of nuclear compartments by either penetrating these compartments or directing in them proteins encoded by the viral genome (*Fig. 1*). Although viruses also interact with other nuclear compartments, the process of viral interaction with the nucleolus and PML bodies has been the most thoroughly studied. Along with this, new compartments assemble in the nuclei in which viruses replicate. All of these processes are discussed in more detail below.

The nucleolus is the most recognizable functional compartment of the cell nucleus. The main function of the nucleolus is ribosome biogenesis. However, the nucleolus also has a series of other, so-called non-canonical, functions. It acts as a site for the sequestration of various proteins and participates in cell cycle regulation, response to stress, organization of the repressive genome compartment, and in a number of other functional processes [37]. Thus, it is not surprising that viruses interact closely with the nucleolus during an infection. This applies to both the viruses replicating in the cell nucleus and those replicating in the cytoplasm. The result of the interaction mediated by the transfer of various viral proteins to the nucleolus can be either complete/partial disintegration of the nucleolus, relocalization of nucleolar proteins to the nucleoplasm and cytoplasm, or relocation of nucleoplasmic proteins to the nucleolus [38-42].

Early studies have shown that the effectiveness of the infectious process directly depends on the interaction between the virus and the nucleolus [43, 44, 45]. With the development of proteomics, more complete data on the spectrum of viral and nucleolar proteins that interact with each other have been obtained [41, 46-49]. Experiments comparing the proteome of nucleoli isolated from healthy cells and cells infected with adenovirus suggest that movement from the nucleolus or into the nucleolus involves a very wide range of proteins [39, 40, 41, 50, 51]. Typical nucleolar proteins are relocated to the viral replication centers (see section 3), the nucleoplasm, and the cytoplasm. Both viral proteins and a number of cellular proteins move to the nucleolus. However, the consequences of this relocation are not always clear. The interactions between viruses and the nucleolus result from the superposition of two diametrically opposed processes: (1) cellular antiviral strategy and (2) viral strategy aimed at evading the antiviral response and maximizing the use of available cellular resources for its own purposes.

The role of nucleolin in antiviral protection has been rather fully characterized. However, it remains unclear whether the release of nucleolin from the nucleolus correlates with the implementation of its antiviral properties. Moreover, in addition to the nuclear protein nucleolin, which mainly resides in the nucleolus, the cell contains cytoplasmic nucleolin and plasma membrane-associated nucleolin [52, 53]. In some cases, it remains unclear which pool of nucleolin is used in antiviral defense. When cells are infected with a highly pathogenic strain H5N1 of the influenza virus, nucleolin expression inhibition significantly increases the activity of viral polymerase. It also enhances the synthesis of viral mRNA, as well as apoptosis and necrosis of the host cell. On the contrary, overexpression of nucleolin decreases infection intensity [54]. Antiviral activity of nucleolin has also been demonstrated in the infection of cells with the goat plague virus (peste des petits ruminants virus, PPRV). This activity is associated with the induction of the host interferon response [55]. Binding of nucleolin to G-quadruplexes in viral RNA [56] and DNA [57] inhibits the viral functions, apparently by blocking the promoters [57].

Apoptosis induction in infected cells is considered one of the mechanisms of the body’s defense against an infection. In this context, it is worth mentioning that one of the elements of the host antiviral defense is sequestration of viral anti-apoptotic factors in the nucleolus and the release of cellular pro-apoptotic factors from the nucleolus. For instance, the PICT-1 protein binds to the apoptosis inhibitor KS-Bcl-2 of Kaposi’s sarcoma-associated herpesvirus (KSHV) and inhibits its anti-apoptotic activity by sequestering KS-Bcl-2 in the nucleolus [58].

The specific mechanisms of induction of nucleolar stress and apoptosis upon penetration of the virus into the cell and the possibilities of reprofiling of these processes for viral reproduction are not always clear. There are many studies demonstrating the complex nature of the interaction between viral proteins and nucleolar components. For instance, the NS protein of the Schmallenberg virus induces a disruption of the nucleolus and relocalization of nucleophosmin from the nucleolus to the nucleoplasm [59]. Poliovirus protease 3Cpro, which is targeted to the nucleolus, modifies UBF and SL1 involved in rDNA transcription and cleaves the transcription factor TAF110, thus inhibiting the synthesis of ribosomal RNA (rRNA) [60]. Precursors of the human rhinovirus 16 protease 3Cpro co-localize with nucleophosmin in the nucleolus. This triggers the cleavage of the OCT-1 transcription factor and complete arrest of the transcription of cellular DNA [61]. The human immunodeficiency virus (HIV) protein Tat interacts with fibrillarin and U3 small nucleolar RNA (snoRNA), resulting in impaired rRNA maturation [62]. The NS1 protein of the influenza virus H3N2 interacts with NOLC1, which regulates rDNA transcription by binding to the large subunit of RNA polymerase. This interaction reduces NOLC1 levels, which leads to apoptosis [63]. Association of the same protein with nucleolin causes hypermethylation of the UCE (upstream control element) of rRNA genes, arrest of rRNA synthesis, and subsequent nucleolar stress [64]. The opposite process, such as the activation of rRNA gene transcription, can be observed when cells are infected with other viruses and an alternative course of the infection (latent infection) takes place. The core protein of the hepatitis C virus binds to nucleophosmin and relocates to the nucleolus, where it interacts with UBF and RNA polymerase I. This interaction enhances the association of these factors with the rRNA gene promoters and increases the level of rRNA transcription. The nucleolus grows in size and moves to the periphery of the nucleus [65]. The HBx oncoprotein of the hepatitis B virus acts in a similar way. HBx is transported to the nucleolus by nucleophosmin and acetylates nucleophosmin, which results in depletion of histones from the rDNA promoters. This, in turn, enhances the transcriptional activity of the nucleolus and the proliferative activity of the cell [66]. In combination with other mechanisms of proliferation control [67], chronic infection leads to cell transformation. The significance of all these observations in the context of viral strategy and the mechanisms of antiviral defense are yet to be elucidated.

Along with evading the antiviral response, viruses actively exploit the proteins sequestered in the nucleolus for their own purposes. In some cases, viruses also use the nucleolus as a compartment partially isolated from the nucleoplasm. During the infection, proteins of the nucleolus can be directly adopted for replication and transcription of viral nucleic acids, as well as the assembly of viral particles. Viruses with a negative-strand RNA genome (influenza virus, Thogotovirus, and Borna disease virus) replicate genomic RNA in the nucleus and closely interact with the nucleolus. Early studies showed that the Borna disease virus uses the nucleolus as a replication site [68]. The positive strand of the hepatitis delta virus RNA is transcribed in the nucleolus, while the negative strand is synthesized in the nucleoplasm [69]. Such segregation allows the virus to exploit the transcriptional machinery and compartmentalization of the host cell nucleus to its maximum efficiency. In the case of a human immunodeficiency virus (HIV-1) infection, the nucleolus is the site of assembly of the complexes providing transport of unspliced and partially spliced viral RNAs to the cytoplasm. Unspliced HIV-1 RNA acts as both genomic RNA and mRNA for the synthesis of Gag and Gag-Pol proteins. Incompletely spliced RNAs act as mRNA for the synthesis of the Vif, Vpr, Tat, Vpu, and Env proteins. Fully spliced RNAs are mRNA templates for the synthesis of the Vpr, Tat, Rev, and Nef proteins. Unspliced and incompletely spliced HIV-1 RNAs are unstable and rapidly degrade in the nucleus. The Rev protein protects these RNAs from degradation and ensures their transport to the cytoplasm. Such an intricate transport complex is formed in the nucleolus to which unspliced and partially spliced HIV-1 RNAs are relocated. Rev is synthesized in the cytoplasm from a spliced RNA and contains signals of nuclear and nucleolar localization. After being transported to the nucleus, Rev associates with nucleoporins Nup98 and Nup214, as well as with the exportin CRM1. The resulting complex is then transported to the nucleolus [70, 71, 72], where Rev multimerizes and binds to specific RRE sequences in the viral RNA [73]. Thus, in the course of an infection, the virus uses both the host cell proteins and the nucleolus as a “staging post” and a platform for the assembly of viral RNPs.

However, a more common phenomenon is the virus-induced relocalization of nucleolar proteins to the nucleoplasm with their further use for viral replication. Viral replication compartments (see section 3 and *Fig. 1*) contain various nucleolar proteins: nucleophosmin, nucleolin, fibrillarin, UBF, Nopp140, POLR1A, TCOFI, and NOLC1 [74, 75, 76]. The structure and protein composition of the nucleolus are significantly altered in cells infected with herpes viruses (HSV-1 and HCMV) [38]. The three main nucleolar proteins, namely, nucleolin, nucleophosmin, and fibrillarin, as well as RPA194, move to the virus replication compartments. There, they participate in the replication, transcription, and assembly of viral particles. A number of studies have shown that nucleolin is involved in the formation of the replication compartments of various herpes viruses [38, 42]. In combination with the viral nuclease UL12, nucleolin is responsible for the maturation of the viral genome and nucleocapsid release from the nucleus [77, 78]. In a cytomegalovirus infection, association of nucleolin with the viral DNA polymerase component UL44 is necessary for efficient DNA replication and the expression of late proteins [79].

In an infection with the influenza virus, accumulation of the multifunctional viral protein NS1 in the nucleolus is accompanied by the delocalization of nucleolin to the nuclear periphery and redistribution of fibrillarin [80]. Nucleolin is believed to ensure the transport of ribonucleoprotein complexes and participate in viral RNA replication. The nucleolar protein RRP1B, which is involved in ribosome biogenesis, relocates from the nucleolus to the nucleoplasm. There, it associates with RNA-dependent RNA polymerase, thus enhancing the transcription of viral RNA [81]. One of the multifunctional nucleolar proteins, LYAR, moves to the nucleoplasm and cytoplasm from the nucleolus and facilitates the assembly of the ribonucleoprotein complexes of the influenza A virus [82].

Summarizing the above mentioned, one can conclude that viruses can both directly affect the ribosomal gene transcription machinery and modify the protein composition of the nucleoli, as well as use the nucleolus as a safe site for the biogenesis of new viral particles. Thus, a viral infection can affect the homeostasis of the nucleolus, as well as its morphology and compartmentalization. This, in turn, can be used to implement the most effective strategies for pathogen survival and reproduction.

**Repair foci**

Repair foci (DDR foci, DNA damage response) are exploited by many viruses as a source of enzymes for viral replication. These viruses include various parvoviruses, and MVM in particular. After penetrating the cell nucleus, MVM DNA preferentially localizes near the damaged regions of the cellular genome, which are associated with phosphorylated histone H2AX and repair factors [83, 84]. Viral replication centers form near the DDR foci. These centers recruit the DNA polymerases present in the DDR foci and other enzymes involved in viral replication. In the course of the infection, the number of pre-existing DDR foci proves insufficient for the assembly of new viral replication centers. For this reason, the virus stimulates the introduction of new DNA lesions, thus increasing the number of DNA repair foci to be exploited by the virus [84, 85]. Other parvoviruses apparently use a similar mechanism [86-88]. DDR activation is also typical of infection with viruses belonging to some other families [89, 90]. For instance, it has been established that, after penetration of the cell, human papillomavirus localizes at chromosomal fragile sites [91].

**Transcription factories, speckles, and paraspeckles**

Transcription of the genes of DNA viruses is carried out by cellular RNA polymerase II. A significant part of the RNA pol II molecules are sequestered in transcription factories [11, 32, 35, 36, 92, 93, 94]. It remains unclear what transcription factories are. According to some data, stable clusters of RNA polymerases are present in the cell regardless of active transcription. There also exists a different point of view, according to which initiated transcription complexes are assembled into clusters (see [35] for a review). In any case transcription factories are associated with the active compartment of the genome. Most viruses entering the cell nucleus preferentially interact with this very genomic compartment. Virus replication centers are assembled at subsequent stages of the infection (see section 3). It is not entirely clear whether these centers capture pre-existing transcription factories or free RNA polymerase relocates to them as the transcription factories disintegrate. A significant part of the pre-existing transcription factories are ultimately lost, while RNA polymerase II accumulates in the centers of viral replication/transcription [95-98].

Speckles are compartments where the splicing machinery is located [8, 9]. However, there is no clear information on whether these compartments simply offer storage sites for splicing factors, which are recruited to transcription sites as required, or whether splicing can occur directly in speckles [99, 100]. A viral infection leads to speckle reorganization [101, 102, 103]. The early stages of lytic infection are characterized by the redistribution of splicing factors (SC35, SON, SRp20, etc.) to the centers of viral replication/transcription [102-105] (see section 3 and *Fig. 1*). At the later stages of a lytic infection, speckles combine into larger compartments. Spliced viral transcripts can be found in these compartments [106, 107]. Fusion of speckles into larger compartments is typical of the cellular response to various stresses, including a virus infection [108, 109]. The fact that spliced transcripts concentrate in speckles at late stages of an infection suggests that accumulation of these transcripts is one of the stages in their transport to the cytoplasm [106]. A completely different picture emerges for the infection of permissive cells by the influenza virus. Splicing of one of the viral RNAs takes place in speckles [110].

In many cells, small compartments formed on the basis of non-coding RNA NEAT1 are localized next to speckles. These compartments are called paraspeckles [111]. The functions of paraspeckles are not entirely clear. They include sequestration of the RNA-editing adenosine deaminase and stress response [111, 112, 113]. The level of NEAT1 RNA and the number of paraspeckles increase significantly in case of a virus infection [114-117]. Apparently, this occurs due to the activation of the innate immunity, since NEAT1 RNA binds a repressor that inhibits transcription of genes encoding several cytokines, including interleukin-8 [114, 118]. However, one of the studies reported that the herpes simplex virus (HSV-1) adopts the proteins sequestered in paraspeckles for its replication [117]. The research has demonstrated that, during a lytic infection, the HSV-1 genome is localized in paraspeckles and that suppression of NEAT1 reduces the production of viral particles.

**PML bodies**

It has long been known that, at the initial stages of a viral infection, virus-specific proteins are recruited to PML bodies to stimulate their disintegration [119-123]. PML bodies contain numerous proteins. The most char acteristic components among them are PML, hDaxx, ATRX, and Sp100. All these proteins play an important role in non-specific antiviral immunity [124-127], which the virus must inactivate. Different viruses solve this problem in different ways. For instance, the HSV-1 ICP0 protein targeted to PML bodies is a ubiquitin ligase that selectively ubiquitinates SUMOylated proteins, including PML and Sp100. Such modification of the proteins stimulates their degradation by the proteasome system [128, 129]. The cytomegalovirus early protein IE1 suppresses PML SUMOylation, which is critical for the formation of PML bodies [130]. In both cases, the final result is the disintegration of PML bodies. Adenovirus early proteins also relocate to PML bodies and cause DAXX degradation and PML redistribution [131, 132, 133]. Disintegration of PML bodies also occurs during lytic infection of cells by other DNA viruses [134].

It should be noted that, after entering into the nucleus, the genomes of many viruses localize next to the PML bodies [135, 136]. The reasons why this happens are not entirely clear. It is also unclear whether viral genomes are transferred to the pre-existing PML bodies, or new PML bodies are formed close to the viral genomes [137, 138]. In the latter case, the assembly of PML bodies next to the viral genomes can be one of the stages of antiviral defense. The situation can be even more complicated. The virus may require a number of proteins sequestered in PML bodies, including the ubiquitination machinery. It has recently been shown that the adenovirus DNA-binding protein E2A is SUMOylated by the enzymatic machinery of the host cell and recruits the transcription factor Sp100A to viral replication centers. Sp100A is released from PML bodies after PML redistribution from bodies to tracks induced by another viral protein (E4orf3) [139]. Human cytomegalovirus proteins IE1p72 and IE2p86 are transiently localized in PML bodies, where they are SUMOylated [140].

## ASSEMBLY OF NEW COMPARTMENTS: VIRAL REPLICATION CENTERS

A characteristic feature of a lytic infection with DNA viruses is the formation of a new type of functional compartments in the cell nuclei: viral replication centers (VRCs). These centers are assembled around individual viral genomes that have penetrated the cell nucleus and serve as sites of transcription and clonal replication of viral DNA [74, 141]. At the late stages of the infection, each VRC contains numerous copies of viral DNA. All these copies are replicas of the original viral DNA molecule around which the VRC is assembled [142, 143, 144]. Furthermore, areas of active replication and transcription within the VRC can be spatially segregated [145]. The protein composition of VRC is rather complex; it includes both virus-specific and cellular components [74, 141]. The latter include mainly DNA replication enzymes, RNA polymerase II, components of the transcription machinery, a wide range of repair enzymes, and chromatin remodeling factors [49, 146, 147].

At least for the herpesvirus infection, it has been shown that VRCs can change their location within the cell nucleus. During the late stages of the infection they can get fused, which makes recombination between the viral genomes replicated in different VRCs possible [144]. Relocation of VRC within the nucleus is an active process, since it is suppressed by actin and myosin inhibitors. VRCs approach speckles as a result of directed relocation. This, apparently, facilitates the splicing of viral transcripts [107]. It was also shown that, during the lytic Epstein–Barr virus infection, the proteins SC35, SON, SRp20, as well as some other splicing machinery components, relocate from speckles to specific structures on the VRC surface [104]. Thus, the strategies for splicing of viral transcripts may vary for different herpes viruses.

## MODIFICATION OF THE 3D GENOME IN A LYTIC AND LATENT INFECTION AND VIRAL GENOME INTEGRATION

**Lytic infection: preferential association of viruses with the A compartment of the genome and an expansion of the A compartment during the later stages of the infection**

In recent years, a number of studies have focused on the potential existence of regions in the host cell genome with which the virus preferentially interacts at various stages of the lytic infection. All these studies used the approaches based on the ligation of spatially proximal DNA fragments in fixed nuclei (the so-called C methods [156, 157]). By using experimental protocols that allow for the identification of the entire range of contacts between the viral and the host cell genomes, it was shown that viruses preferentially contact the active (A) genomic compartment during a lytic infection [158, 159]. Within the A compartment, adenoviruses preferentially come into contact with any promoters or enhancers [159] while the hepatitis B virus interacts with CpG islands [158]. The Epstein–Barr virus was shown to preferentially come into contact with inactive chromatin during a latent infection [160, 161] and relocate to the active chromatin compartment after induction of viral replication [161]. Association with active chromatin is also characteristic of the influenza virus, which is an RNA virus that replicates in the cell nucleus [162]. The expansion of the A compartment is stimulated by this virus and adenoviruses during a lytic infection. The mechanism of this phenomenon has been revealed for the influenza virus. The virus-specific NS1 protein prevents termination of the transcription of cellular genes at polyadenylation sites. As a result, transcription continues for significant distances beyond the gene (sometimes more than 100 kb). The authors showed that active RNA polymerase promotes cohesin removal from the CTCF-binding sites, thus leading to the loss of chromatin loops and significantly changing the genomic configuration. In addition, the enzymes associated with transcribing RNA polymerase can promote chromatin remodeling by removing repressive marks [162]. The benefits of expanding the A compartment for the virus remain to be explored. Profound inhibition of transcription termination at the gene termini also occurs in a lytic infection caused by the herpes simplex virus [163, 164]. Active chromatin is expanded to the previously inactive regions. However, it is still difficult to draw a conclusion as to how significant expansion of the active chromatin compartment in a herpesvirus infection is. This is because the effect of the infection on genome compartmentalization has not been studied yet for this virus using the Hi-C method.

**Modification of the 3D genome of the host cell during a latent infection guided by viral transcription factors**

**Fig. 2:**
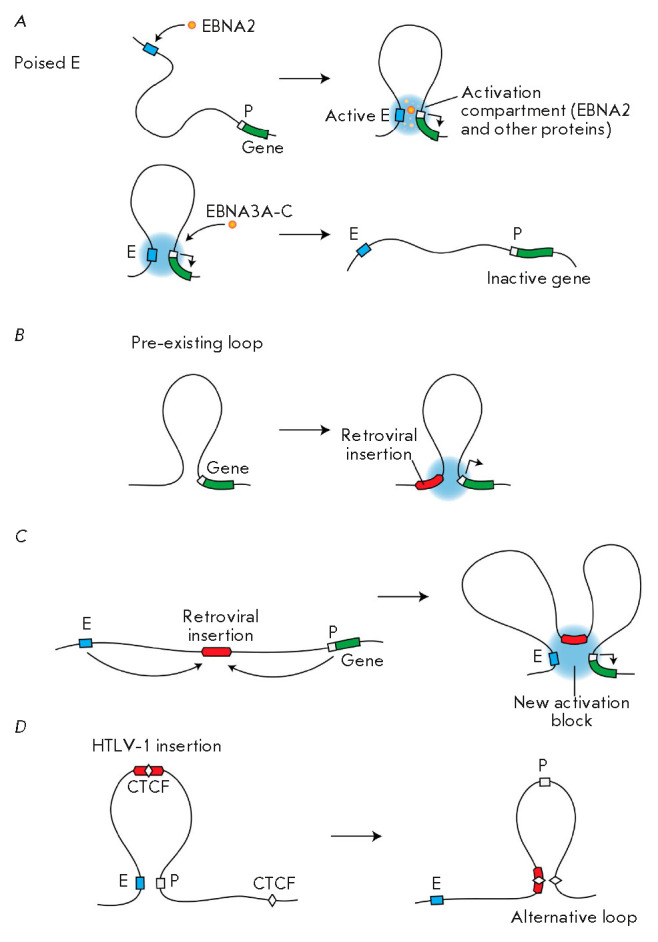
Virus-induced reorganization of the 3D genome. (*A*) Induction (top) or destruction (bottom) of the promoter-enhancer contacts triggered by the viral proteins belonging to the EBNA family with the concomitant activation or repression of the host gene. (*B*) Involvement of the pre-existing genome architecture in the activation of a gene located at a considerable distance from the site of retroviral integration into the genome. (*C*) Formation of a new activator unit via the recruitment of the enchanter and promoter to the site of retroviral integration followed by activation of the host gene transcription. (*D*) Disruption of the promoter-enhancer communication as a result of the introduction of the CTCF-binding site and formation of an alternative loop. P – promoter; E – enhancer

As mentioned above, the Epstein–Barr virus can both cause a lytic infection and reside in cells in latent form as a circular episome associated with chromatin. There are several types of latent infections. They differ in the range of expressed viral proteins [165]. A latent infection with the Epstein–Barr virus is associated with various oncological diseases [166, 167]. For this reason, the mechanisms of epigenetic reprogramming by virus-specific proteins and microRNAs are being intensively studied. The virus-specific protein EBNA2 was shown to associate with enhancers and to modulate the expression of cellular genes by reconfiguring the spatial organization of the genome [168] (*Fig. 2A*). More specifically, EBNA2 activates the transcription of a number of genes, including *c-myc*, by stimulating the emergence of new enhancer-promoter loops [168, 169]. Activation of *c-myc *transcription leads to cell transformation. As a result of such transformation, the cells acquire the ability to unlimitedly proliferate. EBNA3A,C initiate the repression of a specific group of genes, including pro-apoptotic ones. These virus-specific proteins also bind preferentially to enhancer elements [169, 170]. In a number of cases, they prevent the establishment of enhancer-promoter contacts (anti-looping) (*Fig. 2A*). In other cases, EBNA3A,C initiate the assembly of repressive chromatin hubs. These repressive hubs form by recruiting Polycomb repressive complexes [169, 171].

The HIV-1 transcriptional regulator Tat can penetrate any cells via the cell penetration domain (CPD) [172]. Tat is secreted into the blood by T lymphocytes infected with HIV-1 and, once it has entered human B cells, it changes the mutual positions of several genes within the nucleus [173].

It remains difficult to say how widespread the mechanisms of 3D genome reorganization by viral transcriptional regulators are. This issue definitely deserves further study.

**Modification of the 3D genome during integration of viral DNA into the host cell genome**

The problem of insertional mutagenesis caused by the integration of retroviruses into the genome of the host cell is widely being discussed [174-178]. The discussion typically centers on the damage to the genes or the stimulation of the transcription of the cellular genes that have fallen under the control of viral promoters and enhancers [177, 179]. We suggest considering this issue in the context of the 3D genome organization.

First of all, it is worth mentioning that, after integration in the genome, viruses can use the pre-existing genomic architecture to activate the transcription of the host’s distant genes [180] (*Fig. 2B*). This mechanism has been shown, in particular, in the activation of the cyclin D1 (*Ccnd1*) gene by retroviruses integrated into the genome at a considerable distance (100 and 170 kb) upstream of this gene [180]. Activation of the *c-myb *oncogene by the mouse leukemia viruses (MLVs) integrated into the genome at considerable distances from the promoter of this oncogene is carried out in a similar manner [181]. Studies performed using genome-wide methods of analysis have shown that the preferred sites of genomic integration of various retroviruses causing tumors in mice (the so-called common insertion sites [182]) co-localize with various oncogenes within the nuclear space; i.e., in a 3D genome [183].

However, retroviruses not only exploit the pre-existing 3D organization of the genome, but they also trigger its reconfiguration (*Fig. 2C*). Thus, the occurrence of a *de novo *activator complex has been shown in HeLa cells, which carry multiple copies of the human papillomavirus (HPV) in their genome. This complex contains the *c-myc *promoter, a fragment of the HPV genome integrated at a distance of 500 kb upstream of this promoter, and a region of chromosome 8 at a distance of 3,300 kb from the integrated HPV genome. The integrated HPV genome plays a key role in the formation of this complex, since its experimentally induced deletion leads to the loss of all interactions and abrupt reduction in the level of *c-myc *transcription [184].

Another interesting example of spatial reconfiguration of the genome directed by an integrated virus is related to the retrovirus HTLV-1. The DNA copy of its genome contains a CTCF-binding site [185]. It has been shown that in chromosomes containing an integrated HTLV-1 provirus, numerous spatial contacts arise between this provirus and distant genomic regions, which can be located at a distance of several million base pairs. [186]. The establishment of these contacts correlates with changes in the transcription profile. These changes are complex and cannot be ascribed only to the activation of the genes that spatially interact with the provirus [186]. For this reason, it is worth mentioning that the introduction of new CTCF-binding sites in the genome not only gives rise to new spatial contacts, but also disorganizes the pre-existing system of such contacts. In addition, it can also disrupt the pre-existing enhancer-promoter interactions [187, 188] (*Fig. 2D*). CTCF-binding sites are also found in the genomes of other retroviruses [189]. However, the contribution of their integration into the organization of the genome architecture has not yet been studied.

## CONCLUSIONS

There is a lot of evidence on the interaction between virus-specific proteins and functional nuclear compartments in the scientific literature. In this review, we have focused on the studies that provide a mechanistic explanation for the events occurring with intranuclear compartments that are mediated by viral proteins and associated with the infectious process. Meanwhile, most of the published data do not fall under any specific theory in general. For instance, this concerns the causes for temporary deposition of various viral proteins in the nucleolus and relocation of nucleolar components to the nucleoplasm [39-41]. There has been recent evidence that the transcripts of SINE retrotransposons (aluRNA) located in the nucleolus play an important role in maintaining its structural and functional organization [190, 191]. Other studies have shown that transcription of SINE retrotransposons is activated during cell infection with a number of DNA viruses [192]. The question of whether overexpression of these RNAs has an impact on the nucleolus structure remains open. We can hope that the integrated picture will become clearer as new data are accumulated.

It was not until the past few years that virus-induced changes in the 3D genome structure started to draw researchers’ attention. Considering the limited number of publications on this topic, we can only assume that these changes are part of the viral strategy to regulate the host genome. This assumption certainly needs further investigation. A promising trend is studying the possibility of reconfiguring the 3D genome by means of cellular DNA transcription induced from the promoters of the proviral genomes integrated into the host cell genome [179, 193, 194]. For now, such transcription is analyzed only in the context of the possible activation of the adjacent genes. Meanwhile, intergenic transcription was shown to promote the removal of cohesin from the CTCF-binding sites [162], which obviously results in reconfiguration of the 3D genome.

Another promising area of research is the possibility to modify the profile of chromosome splitting into TADs upon activation of proviral transcription. It is known that activation of transcription of an endogenous retrotransposon may lead to TAD separation [195]. However, it is reasonable to assume that active transcription of proviruses integrated into the cellular genome in the course of a retroviral infection has similar consequences. It is also interesting to continue the study on the modification of the spatial genome organization mediated by viral proteins binding to the regulatory regions of the host cell genome. There is no reason to assume that this phenomenon is typical only to the EBNA proteins of the Epstein–Barr virus for which this effect has been established [169, 170]. New studies on the trends mentioned above, as well as a number of other related areas, will significantly expand our understanding of the mechanisms of cell infection with various viruses.
